# Purified Phlorizin from *DocynIa Indica* (Wall.) Decne by HSCCC, Compared with Whole Extract, Phlorizin and Non-Phlorizin Fragment Ameliorate Obesity, Insulin Resistance, and Improves Intestinal Barrier Function in High-Fat-Diet-Fed Mice

**DOI:** 10.3390/molecules23102701

**Published:** 2018-10-19

**Authors:** Xiao-yu Zhang, Kang Yi, Jiang Chen, Rui-ping Li, Jie Xie, Yan Jin, Xue-ran Mei, Yao-jun Li, Gang Liu, Zhan-guo Wang

**Affiliations:** 1 College of Life Sciences, Sichuan Normal University, Longquan, Chengdu 610101, China; zhangxy2005@126.com (X.-y.Z.); yikang0329@126.com (K.Y.); Liruiping11@163.com (R.-p.L.); xiejaye@163.com (J.X.); lsjy0923@163.com (Y.J.); microLeeYJ@163.com (Y.-j.L.); Gangliu1017@126.com (G.L.); 2 State Key Laboratory for Quality Research in Chinese Medicine, University of Macau, Macao SAR, China; 18328616954@163.com; 3 College of Life Sciences, Sichuan University, Wuhou, Chengdu 610065, China; 251899912@163.com; 4 School of Medicine and Nursing, Chengdu University, Longquan, Chengdu 610106, China

**Keywords:** *Docynia indica* (Wall.) Decne, Phlorizin, Anti-obesity, Intestinal barrier, Interaction, HSCCC

## Abstract

Natural products generally contain complex and multiple bioactive compounds that are responsible for the effects on health through complicated synergistic and/or suppressive actions. As an important raw material of local ethnic minority tea, ethnomedicines and food supplements in southwestern areas of China, *Docynia indica* (Wall.) Decne (DID) mainly consists of phlorizin (PHZ), which is the main active component. In this study, the holistic activities and the interactions of components of PHZ, non-phlorizin (NP) in the DID extract (DIDE) were evaluated. A rapid and effective high-speed counter-current chromatography (HSCCC) was performed to knock out PHZ from DIDE and the purity of PHZ was 96.01% determined by HPLC, with a recovery rate of 96.76%. After 13 weeks of treatment course in a high-fat diet (HFD)-induced obese mice model, the results revealed that the DIDE and PHZ significantly decreased weight gain, blood lipid levels, hyperplasia of adipocytes and alleviated inflammation (*p* < 0.05). Both DIDE and PHZ improves insulin resistance (*p* < 0.001). Meanwhile, the intestinal barrier function was improved compared to HFD group, through the determination of serum lipopolysaccharides (LPS), glucagon-likepeptide-2 (GLP-2) and hematoxylin-eosin staining of jejunum. Interestingly, after NP treatment, the metabolic syndrome of the HFD-induced obesity appeared to have a similar improvement. All the experiments showed that there is a synergistic weakening phenomenon when PHZ and NP interact with each other in the mixed state. In conclusion, for the PHZ and NP showing a good effect on anti-obesity, anti-inflammation, and intestinal barrier function, DIDE could be a good source of functional food to prevent obesity.

## 1. Introduction

Obesity is a metabolic disorder characterized by the disrupted dynamics of energy homeostasis, which depends on excessive energy intake and reduced physical activity [1]. It is closely related to metabolic diseases like insulin resistance, diabetes, hyperlipidemia, hypertension, fatty liver, and cardiovascular disease, which is considered by the World Health Organization to be a global epidemic [2,3,4]. In 2017, the incidence of obesity raised at least seven-fold in the past 40 years [5]. So far, there has not been a very satisfactory and safe weight loss drug, and other weight loss methods also have the drawbacks of reducing body immunity, toxic side effects, and easy rebound [6,7]. Dietary supplementation of natural substances with antioxidant activity was now regarded as a safe and effective strategy for the prevention and treatment of obesity, which could improve the development of safe, green, and natural active substances instead of medicine [8,9].

*Docynia indica* (Wall.) Decne (DID) is an evergreen belonging to the *Rosaceae* family, which distributes widely in southwest China and southeast Asia [10]. The DID leaves were widely used as tea or a drug for the healing of fever, cancer, empyrosis, fracture, and rheumatic disease by the local ethnic minorities in southwestern China, with lipid-lowering and weight-loss effects [11,12,13,14]. The DID fruits were consumed by the locals. Existing studies have demonstrated that the content of polyphenols and flavonoids in DID was much higher, which exhibited significant bioactivities, including antioxidation, antitumor, anti-obesity, and anti-bacterial activities [15]. Epidemiological studies showed that polyphenols have physiological activities to be resistant to oxidation and to regulate lipid metabolism [16,17]. At present, DID is widely cultivated in Lancang, Yunnan Province, China, mainly for fresh fruit freshening and fruit processing. But the development for DID is still in infancy at present, especially the leaves, and there is no in-depth research on DID leaves. It could be a widely used and cheap source of natural products with a high value. Our previous research has shown that the main polyphenols in DID is phlorizin (PHZ), with content much more than 40% in leaves [18,19], which was the first specific competitive inhibitor of sodium−glucose symporters (SGLTs) located in the mucosa of the small intestine (SGLT1) and the proximal renal tubule of the kidney (SGLT2), and was excellent for the treatment of type 2 diabetes [20,21]. Findings from Shin et al. suggested that supplementation of PHZ could ameliorate obesity in high-fat diet (HFD)-induced obese mice [22]. This indicates that DID leaves may have a good activity based on its high content of PHZ. However, extracts from natural materials always contain complex and multiple constituents that are responsible for their health effects through complicated synergistic and/or suppressive actions. Therefore, in order to elucidate their efficacy, which are required to pay attention to the relationship among the interaction of multiple bioactive compounds [23]. To fully explain the functional components of the DID extract (DIDE) and their interaction, it is necessary to compare and analyze the functions of DIDE, PHZ, and the part of DIDE removed PHZ.

In this study, the PHZ was knocked out from DIDE by high-speed counter-current chromatography (HSCCC), which has been successfully applied for the preparative separation of various natural products [24,25] and the rest of the non-phlorizin (NP) was collected. Then, the activities of DIDE, PHZ, and NP on ameliorating obesity, regulating insulin resistance and inflammation, and improving intestinal barrier function were investigated through HFD-induced obese mice model. By comparison of the effect of PHZ in a mixed state and a monomer state under the same intragastric dose, combined with the role of NP part, functional factors of weight loss in DID were confirmed, which may provide data to support for the ensuing full development and utilization of DID.

## 2. Results

### 2.1. Selection of the Two-Phase Solvent System and Other Conditions of HSCCC

According to the literature [26,27], the partition coefficient (*K*) usually should be in a suitable range of 0.5 to 2.0. In this study, a series of experiments were performed to select the two-phase solvent system for HSCCC separation. The *K*-values of PHZ in each two-solvent system were summarized in Table 1. The results indicated that the solvent systems composed of n-hexane/ethyl acetate/methanol/water (1:4:1:5, *v*/*v*) reflected best *K* values and the best effect of separating PHZ. 

Several additional HSCCC optimization parameters were also examined, such as injection concentration (20, 10 and 5 mg/mL), flow rate (10, 8 and 5 mL/min), and injection volume (5, 2.5 and 1 mL). The results indicated that the injection concentration at 10 mg/mL, flow rate of the mobile phase at 8 mL/min, and injection volume at 2.5 mL were appropriate for deployment in this HSCCC separation.

### 2.2. HSCCC Separation Procedure

**Figure 1:**
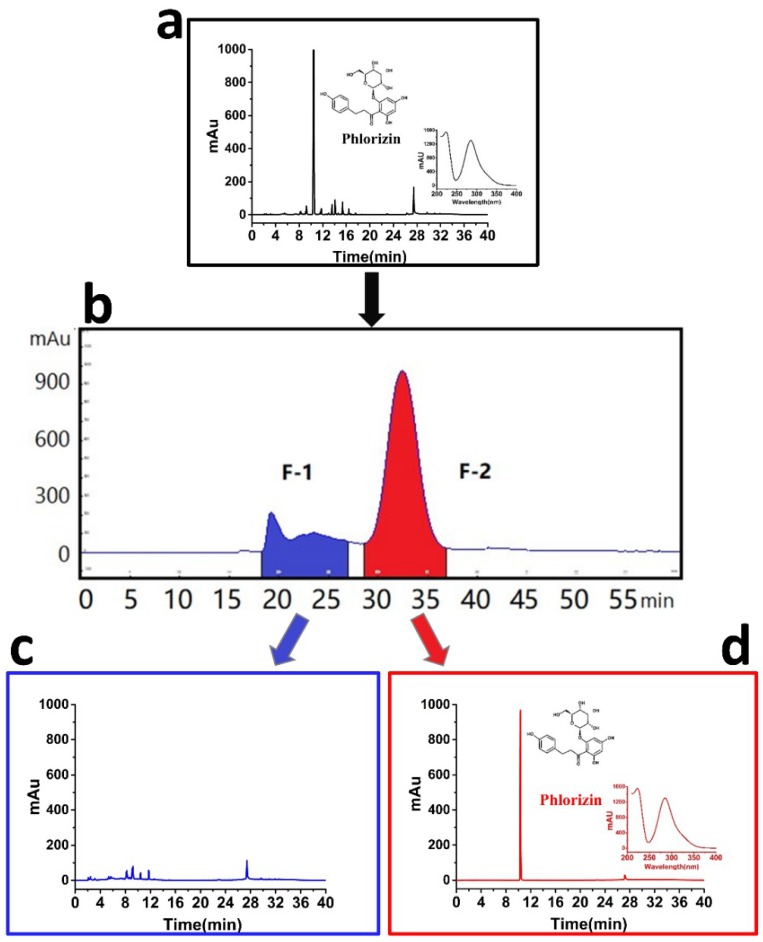
High-speed counter-current chromatography (HSCCC) and HPLC chromatogram. (**a**) HPLC chromatograms of Decne extract (DIDE), (**b**) HSCCC chromatograms of DIDE, (**c**) HSCCC peak fraction 1, (**d**) HSCCC peak fraction 2.

In the present work, 275.0 mg of DIDE powder was rapidly and conveniently separated by HSCCC using the above solvent system after several separations. The chromatogram for the sample separation was shown in Figure 1. After HSCCC separation, peak fraction 1 (F-1) and peak fraction 2 (F-2) were collected and evaporated to dryness under reduced pressure. The compound from F-2 was confirmed to be PHZ by HPLC with its retention time and UV Spectrum compared to the standard. 120.4 mg of PHZ was yielded with a high purity of 96.01% and a high recovery rate of 96.76%. The content of PHZ in DIDE and NP was determined to be 43.43% and 0.51%, respectively (Figure 1a,c).

### 2.3. Effects of DIDE, PHZ and NP on Body Weight, Fat Index, and Fat Cells

**Figure 2:**
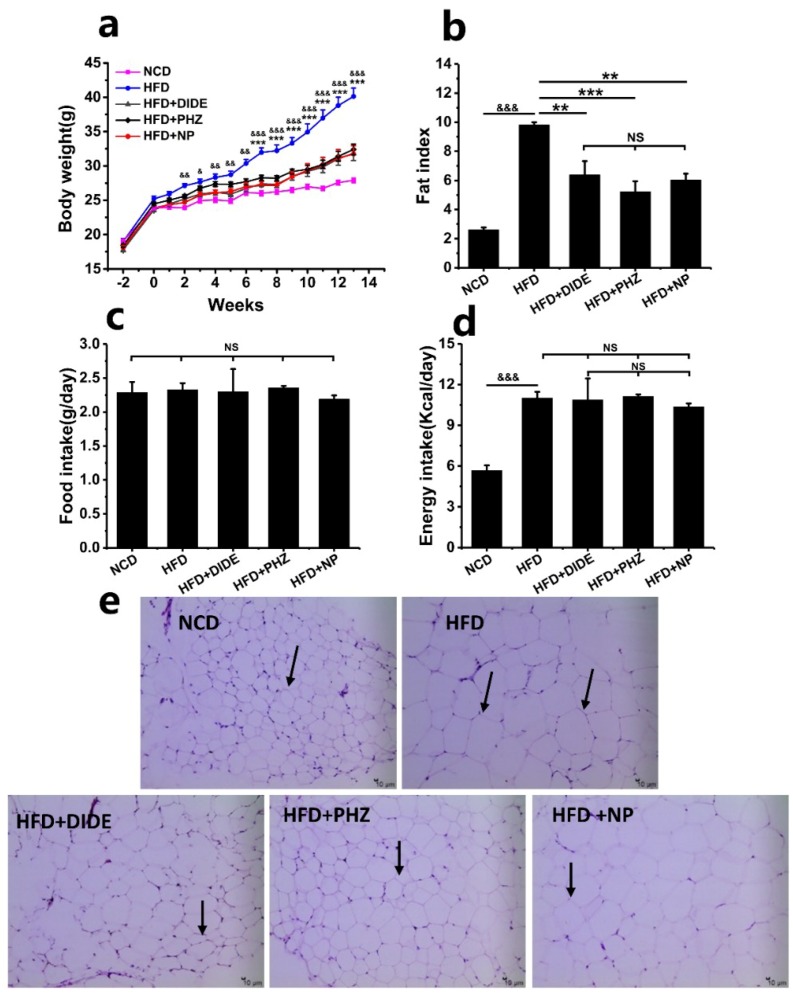
Effects of DIDE, PHZ, and NP on body weight, fat index, food and energy intake, and adipose tissue morphology in high-fat diet (HFD)-fed mice after 13 weeks (n = 10 per group). (**a**) Body weight. (**b**) The index of adipose tissue/100 g B.W, B.W: body weight. (**c**) Food intake. (**d**) Energy intake. (**e**) epididymal adipose tissue morphology. Data are Mean ± SEM. Significant differences between HFD vs. ND are indicated: ^&^ *p* < 0.05; ^&&^ *p* < 0.01; ^&&&^ *p* < 0.001. Significant differences between DIDE, PHZ and NP vs. HFD are indicated: * *p* < 0.05; ** *p* < 0.01; *** *p* < 0.001. NS indicates no significant difference between the two groups. (**e**) A representative photomicrograph of the epididymal adipose tissue is shown at 200× magnification; Arrow, adipocytes.

During the 13 weeks, the body weight of each group mice was weighed. Compared with NCD, the body weight of each group fed with a high-fat diet increased constantly after 2 weeks, and the increase was significant at 13 weeks (Figure 2a; *p* < 0.001). Obviously, the increase of mice body weight in the three treatment groups was significantly suppressed compared with the model group, while there was no significant difference between the three groups (Table 2). Furthermore, the adipose tissue was taken by dissection after 13 weeks. The fat index was calculated as body fat weight (mesentery, retroperitoneal, epididymal, and perirenal) per 100 g total body weight (Figure 2b; Table 2). Compared with the HFD group, the three intervention groups of DIDE, PHZ, and NP significantly reduced the fat index of mice with no significance between each other. PHZ possessed the best effect (Figure 2b). Meanwhile, through microscopic observation of the epididymal fat cells, the HFD could significantly increase the size of the fat cells compared with NCD, while DIDE, PHZ, and NP treatment significantly inhibited the excessive growth of fat cells (Figure 2e). In addition, further statistics on the food intake revealed no significant differences between the five groups (Figure 2c; Table 2; *p* > 0.05). According to the energy calculation, the energy intake of the four groups with a high-fat diet was nearly twice as high as that of the NCD group (Figure 2d; Table 2; *p* < 0.001). By comparing the indicators from the model group and the treatment group, DIDE, PHZ and NP all inhibit the conversion of energy into fat. Therefore, DIDE, PHZ, and NP could inhibit the lipid accumulation and fat cell hypertrophy of HFD-induced mice without influence on food intake.

### 2.4. Effects of DIDE, PHZ and NP on Serum Lipid Levels

**Figure 3:**
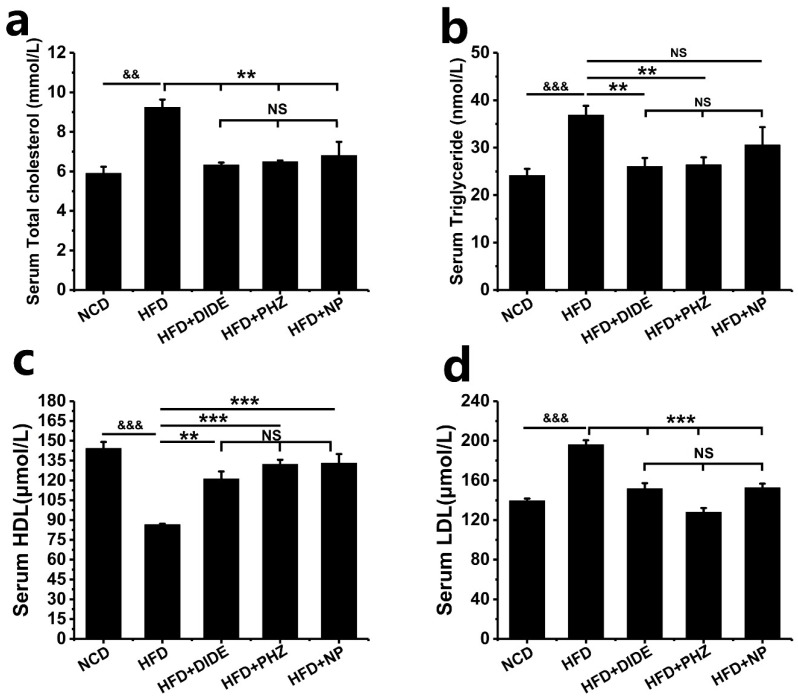
Effects of DIDE, PHZ and NP administration on serum lipids levels (n = 10 per group). (**a**) Serum total cholesterol. (**b**) Serum triglyceride. (**c**) serum HDL (**d**) Serum LDL. Data are Mean ± SEM. Significant differences between HFD vs. ND are indicated: ^&^ *p* < 0.05; ^&&^ *p* < 0.01; ^&&&^ *p* < 0.001. Significant differences between DIDE, PHZ and NP vs. HFD are indicated: * *p* < 0.05; ** *p* < 0.01; *** *p* < 0.001. NS indicates no significant difference between the two groups.

Obesity usually causes abnormal blood lipids. The blood of treated mice was measured at the end of the experiment. Compared with the NCD group, the serum levels of TC (*p* < 0.01), TG (*p* < 0.01), and LDL (*p* < 0.01) in the HFD group are significantly increased, indicating that the high-fat diet causes significant dyslipidemia. Compared with the HFD group, the three treatment groups were able to reduce the levels of TC, TG, and LDL in the serum, and the effects of DIDE and PHZ on reducing TC, TG and LDL were significantly different from those in the HFD group (Figure 3a,b,d; Table 2). However, the effect of NP on TG content is not obvious. The HDL content in serum was determined. Compared with the NCD group, the HDL content in the HFD group was significantly decreased (Figure 3c; *p* < 0.001), while DIDE (*p* < 0.01), PHZ (*p* < 0.01) and NP (*p* < 0.01) were able to significantly increase the serum HDL content, and there was no significant difference between the three groups. The above results indicate that DIDE, PHZ, and NP can improve dyslipidemia caused by high-fat diet and make it normal.

### 2.5. DIDE, PHZ and NP Improves Diet-Induced Insulin Resistance

**Figure 4:**
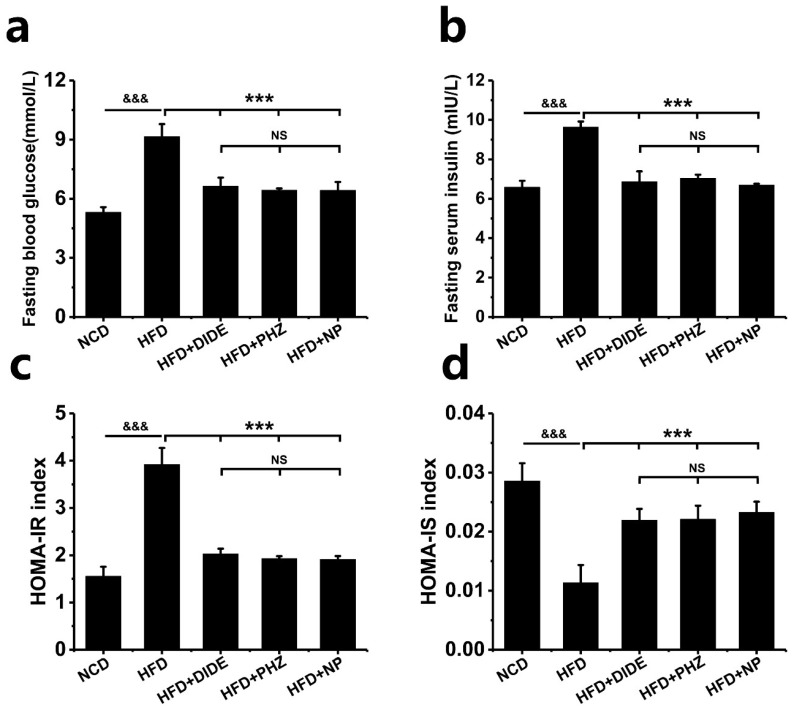
Effects of DIDE, PHZ and NP administration on plasma glycemia markers (n = 10 per group). (**a**) Fasting blood glucose. (**b**) Fasting Serum insulin. (**c**) HOMA-IR index. (**d**) HOMA-IS index. Data are Mean ± SEM. Significant differences between HFD vs. ND are indicated: ^&^ *p* < 0.05; ^&&^ *p* < 0.01; ^&&&^ *p* < 0.001. Significant differences between DIDE, PHZ and NP vs. HFD are indicated: * *p* < 0.05; ** *p* < 0.01; *** *p* < 0.001. NS indicates no significant difference between the two groups.

A high-fat diet induced an anabatic in the fasting serum glucose and fasting serum insulin, consequently an increase of the HOMA-IR and decrease of the HOMA-IS index (Figure 4; Table 2;) occurred, while the mice after the DIDE, PHZ and NP treatment showed a significant decrease in the fasting serum glucose and insulin levels and HOMA-IR index, yet a distinct increase of HOMA-IS index compared to mice in HFD group. This suggests that that DIDE, PHZ, and NP significantly improved high-fat diet-induced hyperglycemia and insulin resistance in mice. There was no significant difference between the groups of DIDE, PHZ and NP.

### 2.6. DIDE, PHZ, and NP Ameliorated HFD-Induced Inflammation Cytokines Levels in Serum

**Figure 5:**
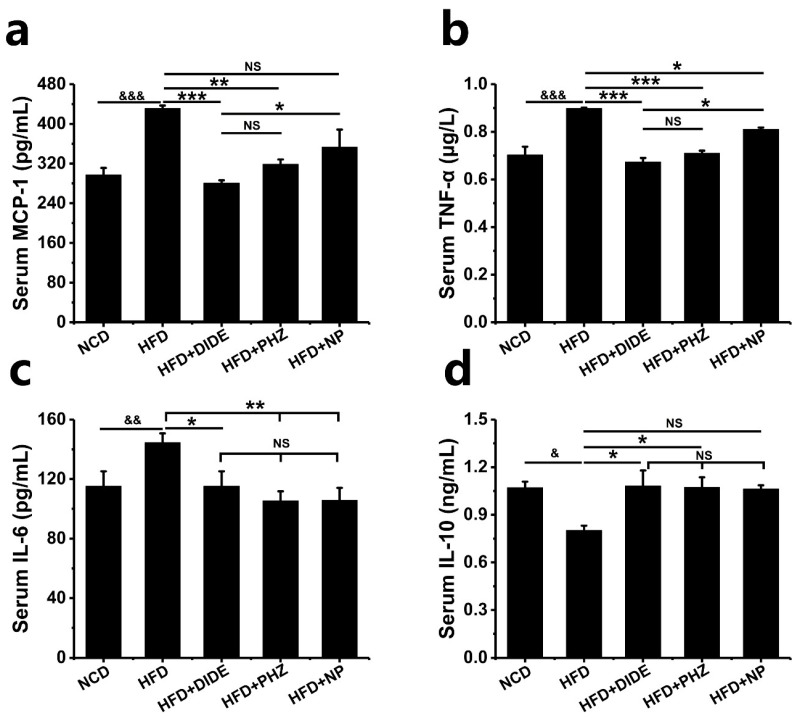
Effects of DIDE, PHZ and NP administration on serum inflammation (n = 10 per group). (**a**) MCP-1; (**b**) TNF-α; (**c**) IL-6; (**d**) IL-10. Data are Mean ± SEM. Significant differences between HFD vs. ND are indicated: ^&^ *p* < 0.05; ^&&^ *p* < 0.01; ^&&&^ *p* < 0.001. Significant differences between DIDE, PHZ and NP vs. HFD are indicated: * *p* < 0.05; ** *p* < 0.01; *** *p* < 0.001. NS indicates no significant difference between the two groups.

Hypertrophic adipocytes can secrete various pro-inflammatory cytokines such as TNF-α and IL-6, and the level of pro-inflammatory factor MCP-1 is believed to be significantly increased during the development and progression of obesity. In the current study, the serum levels of MCP-1 (*p* < 0.001), TNF-α (*p* < 0.001) and IL-6 (*p* < 0.01) in HFD-induced mice groups were significantly increased. It indicated that the metabolic homeostasis and inflammatory response occurred in the obesity caused by a high fat diet. DIDE, PHZ, and NP could reduce the levels of MCP-1, TNF-α, and IL-6 in mice serum, compared with the HFD group (Figure 5a–c; Table 2). Meanwhile, the content of anti-inflammatory factor IL-10 in serum was determined, and the results were improved to varying degrees after three interventions. However, after statistical analysis, the effects of NP on MCP-1 and IL-10 were not significantly different from those in the HFD group (Figure 5a,d; *p* > 0.05). It indicated that DIDE and PHZ are better than NP in improving the inflammatory response in obese mice.

### 2.7. DIDE, PHZ and NP Improve Intestinal Barrier Damage and Reduce LPS Level in the Blood

**Figure 6:**
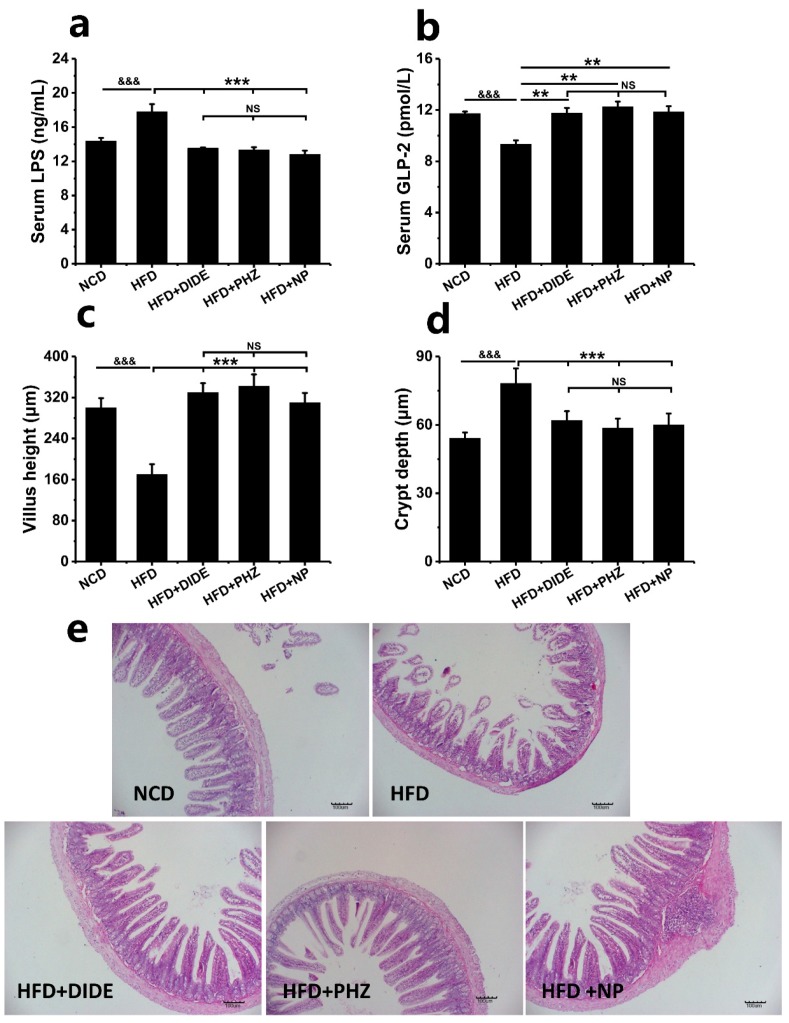
Effects of DIDE, PHZ, and NP on serum LPS, GLP-2 and jejunum barrier function (n =10 per group). (**a**) serum LPS. (**b**) serum GLP-2. (**c**) villus height. (**d**) crypt depth. (**e**) jejunum morphology. Data are Mean ± SEM. Significant differences between HFD vs. ND are indicated: ^&^ *p* < 0.05; ^&&^ *p* < 0.01; ^&&&^ *p* < 0.001. Significant differences between DIDE, PHZ and NP vs. HFD are indicated: * *p* < 0.05; ** *p* < 0.01; *** *p* < 0.001. (**e**) A representative photomicrograph of the jejunum tissue is shown at 200 magnification. NS indicates no significant difference between the two groups.

When the body’s metabolic disorder leaded to obesity, it was always accompanied by changes to the intestinal barrier function [28]. The integrity of tight junctions and adhesive junctions between the intestinal epithelial cells will increase the permeability of the intestinal barrier, which will eventually lead to more enterotoxins such as lipopolysaccharide entering the circulatory system [29]. In this study, the jejunum was selected from the small intestine tissue sections. The tissue structures of each group were observed by HE staining. The results were shown in Figure 6. Compared with the NCD group, the villus height in the jejunum of the HFD group decreased significantly (Figure 6c; *p* < 0.001), and even multiple lesions increased crypt depth (Figure 6e). After DIDE, PHZ, and NP intervention, the villus height was significantly improved and the depth of the crypt was reduced (Figure 6c–e). These data indicated that the three compounds could significantly reduce the damage of intestinal tissue. 

In addition, GLP-2 is a specific enteric trophic factor secreted by ileal L cells. Its increased secretion will enhance the mechanical barrier function of the intestine. The levels of GLP-2 and LPS in the blood of each group of mice were determined and as shown in Figure 6b, the GLP-2 content in the obese mice of the HFD group decreased significantly compared with NCD group (*p* < 0.001). After the intervention, the levels of GLP-2 in the three groups were significantly higher than those in the model group (*p* < 0.01). Serum LPS levels were significantly lower in the three treatment groups than that in HFD group and did not differ from NCD (Figure 6a; *p* < 0.001). It indicated that the intestinal barrier function was obviously impaired after HFD-induced obesity, while DIDE, PHZ, and NP could improve the mechanical barrier of the small intestine by regulating the secretion of GLP-2 and reducing the entry of LPS into the circulatory system through the small intestine to suppress chronic inflammation and insulin resistance.

## 3. Discussion

In this study, a simple, rapid, and efficient method for separation and purification of PHZ by HSCCC was established. The separation time was only 50 min, the purity and yield reached 96.01% and 96.76%, respectively. A similar separation method of PHZ from Sweet Tea was performed by Sun et al. [30], but the separation was performed twice, and the separation time was as long as 280 min. It indicates that the efficiency of the current study was more improved. The total recovery of the sample reached 92.05%, indicating that the HSCCC had a small loss and high recovery rate when separating and purifying the extract. It was suggested that the HSCCC method could be used to knock out different chemical components, which will be a good choice for exploring the functional factors in the total extract.

Previous experiments have shown that DID had a good anti-obesity effect. DIDE was administered intragastrically while the mice were given a HFD in the current study. The results showed that the food intake of the mice was almost unaffected, but the increase in body weight was twice as high as that in the HFD group compared with the normal diet group, indicating that DID can effectively prevent the occurrence of obesity. Under the intervention of DIDE, the weight of the mouse adipose tissue (epididymis, perirenal, and abdominal cavity) was significantly reduced, indicating that mice can significantly reduce the conversion of energy to fat. Studies have found that the increased abundance of *Firmicutes* in gut microbes of obese patients could increase the capacity of harvesting energy from the diet, which promotes more effective calorie absorption and subsequent weight gain [31,32]. Therefore, it was speculated that DIDE may reduce the ability to obtain energy from feed by changing the composition of the intestinal flora and reducing the amount of *Firmicutes*. In addition, according to the physiological effects of animals and the metabolic characteristics of energy substances, the intervention of DIDE may increase the energy consumption of animals, or burn more in the form of heat, or be consumed due to increased exercise. Unfortunately, this experiment did not address intestinal flora, as well as observations of mouse behavior and measurement of caloric forms. Meanwhile, DIDE could reverse the changes of these serum lipids, such as hyperglycemia and insulin resistance altered by the HFD feeding, which correlate with obesity-related metabolic disturbances that include hypertension, insulin resistance, impaired insulin secretion and cardiovascular disease [33,34]. The experiment also found that DIDE increased the content of anti-inflammatory factors IL-10, GLP-2, decreased the content of LPS, and effectively prevented intestinal damage. These indicated that DID could improve the intestinal mechanical barrier function and reduce the occurrence of chronic inflammation by reducing LPS into the blood circulation. A large number of studies have reported that intestinal bacteria were closely related to the production of LPS and GLP-2 [35,36]. The short-chain fatty acids produced by the decomposition of certain intestinal bacteria would greatly stimulate the secretion of GLP-2 by ileal L cells, and GLP-2 would increase the expression of tight junction proteins Octudin-1, Claudin-1 and ZO-1, which enhances the tight junctions between intestinal epithelial cells and the integrity of mechanical barriers [37,38]. Therefore, it is speculated that DIDE may achieve weight loss by regulating the intestinal “flora-barrier” axis and a more complete analysis of the microorganism in the gut is suggested for further studies.

Experiments have confirmed that PHZ is a weight loss function factor for DID. According to the idea of the consistency of effect-equivalents [23,39] the DIDE group dose of 45 mg/kg contained 20 mg of PHZ, which was consistent with the dose of the PHZ group (20 mg/kg). The experimental data showed the consistency of effect equivalents. Briefly, there were no significant differences between the two groups in preventing obesity, regulating blood lipids, reducing inflammation, and improving intestinal damage. The above data strongly confirmed that PHZ was an important functional factor in DID. This was also consistent with the literature that PHZ had the effect of losing weight [22]. In addition, PHZ-rich Sweet Tea extract was also reported to have a weight loss effect and its minimum effective dose was 150 mg/kg [40]. The current DIDE dose was only one-third of that dose, which reveals the great advantage and potential of using DID as a new resource to develop into a weight loss product.

In the study of the function of natural products and foods, integrity cannot be ignored. NP was obtained in this work and the functional evaluation was also based on the effector equivalent-consistency. The dose of the NP was determined to be 25 mg/kg (45 mg DIDE minus 20 mg PHZ). Various experimental data showed that this part of the substance also had the effect of losing weight, regulating blood lipids, reducing inflammation, and improving intestinal damage, even though it was a little worse than DIDE and PHZ in some indicators such as inflammatory factors. Therefore, it could be realized that the functional factors in DID had other substances besides PHZ. According to the chromatogram of the NP part with spectral analysis, it was found that the ultraviolet absorption characteristics of this part were weak, and the water-soluble polysaccharide content reached 20%. It was speculated that this part may have macromolecules such as polysaccharides and dietary fibers. In addition, a significant number of researchers have reported that polysaccharides and dietary fiber had many benefits for obese and diabetic patients [41,42].

Further comparison of the effect equivalents of DIDE, PHZ, and NP revealed that the three relationships at the time of administration were: DIDE (45 mg/kg) = PHZ (20 mg/kg) + NP (25 mg/kg), but the efficacy relationship was: DIDE (45 mg/kg) < PHZ (20 mg/kg) + NP (25 mg/kg), It indicated that there might be a synergistic effect between the components contained in DIDE or the two substances of PHZ and NP interacted with each other in the mixed state, that is a synergistic weakening phenomenon. Functional foods contain complex and multiple constituents that are responsible for their health effects through complicated synergistic and/or suppressive actions. It is needed to pay attention to the relationships between the proportions of components and activity and the interaction of multiple bioactive compounds. Briefly, the active substances in plants, as a “team”, work together to absorb. The body needs different active substances, and each function can achieve balance. Not only the need for these substances, but also the ratio between them is very important. This also suggests that we should fully consider the location of use and the way of use because of the synergy between various substances when developing and utilizing resources, such as refined or simple processing.

In conclusion, DIDE was a good PHZ source plant, which had a good effect on anti-obesity, and NP also had a good potential. Then DID can be used for roughing and refining, and the development form is diverse. This provides a reference for developing DIDE into a functional food that prevents obesity.

## 4. Materials and Methods

### 4.1. Materials

Healthy young leaves of *Docynia indica* (Wall.) Decne were collected from the Pu’er (Yunnan, China) in 2014, which were identified and confirmed by Professor Qiang Luo from College of Xichang (Chengdu, Sichuan, China) according to the plant characteristics of DID [18]. The voucher specimen code is: PE-20141020. All the leaves were dried in open air and kept in a dry environment.

Ethyl acetate, n-hexane, n-butanol, and methanol used for HSCCC separation were analytical grade and obtained from the Kelong Reagent Co., Ltd. (Chengdu, Sichuan, China). The acetonitrile (HPLC grade) were purchased from Tedia Company Inc. (Fairfield, SC, USA). Deionized water (18.25 MΩ) was obtained from a Milli-Q water purification system (Millipore, Bedford, MA, USA). The PHZ standard with purities over 98% was purchased from Chengdu Herbpurify Co., Ltd. (Approval No. Y-029-121123; Chengdu, Sichuan, China).

### 4.2. Preparation of DIDE Powder

The method of DIDE powder preparation referred to Chen et al. [43]. Briefly, 100.00 g of DID leaves powder (60 mesh) were extracted twice with 2.0 L of 60% ethanol by ultrasonic (250 W) for 50 min at 50 °C. All the filtrates were combined and concentrated by rotary evaporation at reduced pressure and then freeze-dried. 35.56 g of DIDE powder were obtained and stored in 4 °C for subsequent HSCCC separation.

### 4.3. Selection of Two-Phase Solvent System

A TBE-300C (Shanghai Tauto Biotech Co., Ltd., 122 Shanghai, China) was coupled with a set of three multilayer coils and a 20 mL sample loop for HSCCC separation. In the present study, three kinds of solvent systems at different volume ratios were used as the two-phase solvent system in HSCCC (ethyl acetate/methanol/water, n-butanol/ethyl acetate/methanol/water and n-hexane/ethyl acetate/methanol/water). The solvent system ratios were selected according to the partition coefficients (*K*) of PHZ. The *K* values of the target components were determined according to the literature [44] by HPLC analysis as follows: about 2 mL of each phase of the equilibrated two-phase solvent system was added to approximately 4 mg of the DIDE. The tube was rigorously shaken for 1 min and static at 25 °C until equilibrium was obtained; then 1 mL of upper and lower phases of solvent system were analyzed by HPLC, respectively, to obtain *K* values of PHZ. The *K* was defined as *K* = *A*_upper_/*A*_lower_, where *A*_upper_ and *A*_lower_ were the HPLC peak area of PHZ in the upper and lower phases, respectively.

### 4.4. HSCCC Separation Procedure

The coil column was first filled with the upper phase of the solvent. While the apparatus was rotated at 850 rpm and then the lower phase was pumped into the column at the flow rate of 8 mL/min in the head end coil column. After the mobile phase front emerged and hydrodynamic equilibrium was established in the column, about 2.5 mL of the sample solution, containing 25 mg of DIDE, was injected into the injection valve and the effluent from the column outlet was monitored at 285 nm. PHZ and NP of HSCCC were collected according to the elution profile and concentrated at reduced pressure (60 °C, −0.08 MPa). The entire HSCCC separation experiment was conducted at 26 °C. 

### 4.5. Determination of Compounds by HPLC

The DIDE, PHZ (standard) and each fraction corresponding to various portions of the major peaks in HSCCC were analyzed by HPLC. HPLC analysis was performed on an Agilent 1200 HPLC system (Agilent, Santa Clara, CA, USA) equipped with an autosampler, thermostat, binary pump, column compartment, and diode array detector (DAD). The HPLC separation was performed on an Agilent Eclipse C_18_ column (4.6 × 250 mm, 5 μm) at 25 °C. The binary gradient consisted of deionized water (solvent A) and acetonitrile (solvent B), following the elution program: 0 min (15% B), 15 min (50% B), 30 min (100% B), 45 min (15% B). A flow rate of 1 mL/min was used, and samples (10 μL) were detected at 285 nm [45]. All solutions were filtered through a 0.45 μm membrane filter before HPLC analysis. System control and data acquisition were performed on the ChemStation for LC 3D systems.

### 4.6. Animals and Diets

Four-week-old male C57BL/6J mice littermates (Chengdu DOSSY Experimental Animals Co., Ltd.; Chengdu, Sichuan, China) were housed in a controlled environment (room temperature 20–22 °C, room humidity 40–60%; inverted 12 h daylight cycle, lights turned on at 8 a.m. and turned off at 8 p.m.) in groups of five mice per cage, with free access to food and water. The mice were kept under observation for 2 weeks prior to the start of the experiments. All of the following animal experimental procedures were approved by the animal ethics committee of Chengdu University. According to the literature and the results of previous research [46,47] the intragastric dose of PHZ was 20 mg/kg [body weight (BW)/day], and the dosage of the DIDE was fixed to be 45 mg/kg (BW/day) based on the content of PHZ contained (45% of PHZ in DIDE: 45 mg DIDE containing 20 mg PHZ and 25 mg NP), the intragastric dose of the NP is 25 mg/kg (BW/day). The mice were then randomly divided into 5 groups: (1) normal chow diet group (NCD, 10% energy from fat, n = 10); (2) high-fat diet group (HFD, 45% energy from fat, n = 10); (3) high-fat diet with DIDE groups (HFD + DIDE, 45 mg/kg (BW/day), n = 10); (4) high-fat diet with PHZ groups (HFD + PHZ, 20 mg/kg (BW/day), n = 10); (5) high-fat diet with NP groups (HFD + NP, 25 mg/kg (BW/day), n = 10). Dosages of the three treatment groups were met: DIDE (45 mg/kg) = PHZ (20 mg/kg) + NP (25 mg/kg). The ingredients and energy densities of the NCD and HFD are listed in Supplementary Tables S1 and S2. DIDE, PHZ and NP were given daily sterile saline solution by intragastric administration. The NCD group and HFD group received the corresponding volume of sterile saline solution. Body weight and food intake were recorded weekly. Gastric irrigation doses were adjusted weekly based on mice’ weights. At the 13th week, after 12 h of food deprivation, blood was collected, and then serum was isolated by centrifugation of 1707× *g* at 4 °C for 10 min. After blood collection, all animals were sacrificed by cervical dislocation and the adipose tissues were promptly removed, rinsed and weighed. Part of the intestine and epididymis fat is preserved in a formalin solution.

### 4.7. Biochemical Analysis

A multiplex detection kit (Yinggong, Inc., Shanghai, China) was used to determine the levels of serum total-cholesterol (TC), triglyceride (TG), high-density lipoprotein (HDL), low-density lipoprotein (LDL), lipopolysaccharide (LPS), glucagon-like peptide-2 (GLP-2), insulin, tumor necrosis factor (TNF-α), monocyte chemoattractant protein-1 (MCP-1), interleukin-6 (IL-6), and interleukin-10 (IL-10). Blood glucose was determined through a glucose meter (Omron Healthcare, Japan) using 1 μL of blood collected from the tip of the tail vein. The homeostasis model assessment (HOMA) was applied to calculate the insulin resistance (HOMA-IR) and insulin sensitive index (HOMA-IS) in accordance with the following formulas: HOMA-IR = [fasting insulin concentration (mIU/L)] × (fasting glucose concentration (mmol/L)]/22.5; HOMA-IS = 1/[fasting insulin concentration (mIU/L)) × (fasting glucose concentration (mmol/L)] [48]. 

### 4.8. Histopathological Analysis

Epididymis fat and intestine sections were removed from five groups of mice and fixed in a buffer solution of 10% formalin, followed by dehydration, embedding in paraffin, and sectioning. Sections of 4 µm were stained with hematoxylin-eosin and Masson’s trichrome. Five random fields from each section were examined and the stained areas were viewed using an optical microscope with a magnifying power of 200×. 

### 4.9. Statistical Analysis

Statistical analyses were performed in the SPSS 20.0 (Chicago, IL, USA), GraphPad Prism 5.01 (San Diego, CA, USA) and Origin Pro 9 (Origin Lab Corporation, Wellesley Hills, MA, USA). Significant differences were evaluated by one way analysis of variance (ANOVA) and the Tukey test. A value of *p* < 0.05 was considered statistically significant. All data were expressed as mean ± standard error of mean.

## Figures and Tables

**Table 1:** The *K* values of phlorizin (PHZ) in different two-phase solvent systems.

Solvent System	Ratio(*v*/*v*)	*K*
Ethyl acetate-methanol-water	4:1:5	4.5
Ethyl acetate-methanol-water	4:2:5	12
*N*-butanol-ethyl acetate-methanol-water	1:1:0.5:2	5.3
*N*-hexane-ethyl acetate-methanol-water	2:3:1:5	0.1
*N*-hexane-ethyl acetate-methanol-water	1:3:2:4	0.2
*N*-hexane-ethyl acetate-methanol-water	1:4:1:5	1.2
*N*-hexane-ethyl acetate-methanol-water	1:6:3:6	2.5

**Table 2:** Effects of DIDE, PHZ, and NP on metabolic parameters in different groups at the end of 13-week HFD feeding.

Parameter	NCD (n = 10)	HFD (n = 10)	HFD + DIDE (n = 10)	HFD+PHZ (n = 10)	HFD + NP (n = 10)
**Body Weight Measurements**					
Body weight gain (g)	4.01 ± 0.38	14.78 ± 2.07 ^&&&^	7.74 ± 1.05 ***	8.19 ± 0.59 **	8.2 ± 1.01 **
Body weight gain rate (%)	16.79	54.99	32.13	33.84	34.87
Relative body weight gain rate (%)	0	38.2	15.35	17.05	18.08
Food intake (g/day)	2.29 ± 0.15	2.33 ± 0.10	2.3 ± 0.33	2.36 ± 0.03	2.19 ± 0.06
Energy intake (kcal/day)	5.67 ± 0.38	11.01 ± 0.46 ^&&&^	10.87 ± 1.57	11.14 ± 0.14	10.35 ± 0.27
**Serum Biochemical Variables**					
TC (mmol/L)	5.91 ± 0.33	9.24 ± 0.39 ^&&^	6.33 ± 0.11 **	6.49 ± 0.06 **	6.81 ± 0.69 **
TG (mmol/L)	24.15 ± 1.38	36.89 ± 1.92 ^&&&^	26.08 ± 1.75 **	26.40 ± 1.56 **	30.59 ± 3.74
HDL (mmol/L)	144.26 ± 4.85	86.60 ± 0.62 ^&&&^	121.24 ± 5.51 ***	132.32 ± 3.30 ***	133.17 ± 6.75 ***
LDL (mmol/L)	139.46 ± 2.18	196.05 ± 4.54 ^&&&^	151.72 ± 5.45 ***	127.90 ± 4.22 ***	152.66 ± 4.08 ***
Insulin (nIU/mL)	6.59 ± 0.33	9.64 ± 0.28 ^&&&^	6.87 ± 0.52 ***	7.04 ± 0.19 ***	6.69 ± 0.07 ***
Serum TNF-α (pg/mL)	0.70 ± 0.03	0.90 ± 0.01 ^&&&^	0.67 ± 0.02 ***	0.71 ± 0.01 ***	0.81 ± 0.01 ***
Serum MCP-1 (pg/mL)	297.38 ± 13.69	431.18 ± 5.78 ^&&&^	280.53 ± 5.78 ***	318.72 ± 9.58 **	353.48 ± 35.01
Serum IL-6 (pg/mL)	115.21 ± 9.98	144.55 ± 6.15 ^&&^	115.21 ± 9.98 *	105.43 ± 6.35 **	105.78 ± 8.30 **
Serum IL-10 (pg/mL)	1.07 ± 0.04	0.80 ± 0.03 ^&^	1.08 ± 0.10 *	1.07 ± 0.06 *	1.06 ± 0.02
Serum LPS (pg/mL)	14.39 ± 0.35	17.82 ± 0.88 ^&&&^	13.54 ± 0.09 ***	13.35 ± 0.31 ***	12.82 ± 0.43 ***
Serum GLP-2 (pg/mL)	11.73 ± 0.15	9.33 ± 0.31 ^&&&^	11.77 ± 0.40 **	12.26 ± 0.41 **	11.85 ± 0.45 **

All data in the table are mean ± SEM. Significant differences between HFD vs. ND are indicated: ^&^ *p* < 0.05; ^&&^ *p* < 0.01; ^&&&^ *p* < 0.001. Significant differences between DIDE, PHZ and NP vs. HFD are indicated: * *p* < 0.05; ** *p* < 0.01; *** *p* < 0.001.

## Supplementary Materials

The following are available online.

## Abbreviations

DID	*Docynia indica* (Wall.) Decne
PHZ	phlorizin
NP	non-phlorizin
DIDE	extract
HSCCC	high-speed counter-current chromatography
HFD	high-fat diet
NCD	normal chow diet
LPS	lipopolysaccharides
GLP-2	glucagon-like peptide-2
SGLTs	sodium−glucose symporters
TC	total-cholesterol
TG	total-cholesterol
HDL	high-density lipoprotein
LDL	low-density lipoprotein
TNF-α	tumor necrosis factor
MCP-1	monocyte chemoattractant protein-1
IL-6	interleukin-6
IL-10	interleukin-10
HOMA	homeostasis model assessment
HOMA-IR	insulin resistance index
HOMA-IS	insulin sensitive index
BW	body weight
