# Supplementary material for: Purified Phlorizin from DocynIa Indica (Wall.) Decne by HSCCC, Compared with Whole Extract, Phlorizin and Non-Phlorizin Fragment Ameliorate Obesity, Insulin Resistance, and Improves Intestinal Barrier Function in High-Fat-Diet-Fed Mice
Source: Molecules. 2018 Oct 19;23(10):2701. doi: 10.3390/molecules23102701 (PMC6222664; doi:10.3390/molecules23102701)
Supplement: Supplementary file 1 [file molecules-23-02701-s001.pdf]

molecules

MDPI

Article

Supplementary Materials

Purified phlorizin from *DocynIa Indica* (Wall.) Decne by HSCCC, compared with whole extract, phlorizin and non-phlorizin fragment ameliorate obesity, insulin resistance and improves intestinal barrier function in high-fat-diet-fed mice

Xiao-yu Zhang[1†], Kang Yi[1†], Jiang Chen[2], Rui-ping Li[1], Jie Xie[1], Yan Jin[1], Xue-ran Mei[3], Yao-jun Li[1], Gang Liu[1], Zhan-guo Wang[4*]

1 College of Life Sciences, Sichuan Normal University, Longquan, Chengdu 610101, China
2 State Key Laboratory for Quality Research in Chinese Medicine, University of Macau, Macao SAR, China
3 College of Life Sciences, Sichuan University, Wuhou, Chengdu 610065, China
4 School of Medicine and Nursing, Chengdu University, Longquan, Chengdu 610106, China
* Correspondence: wangzhanguo@cdu.edu.cn; Tel: +86 28 84617082; Fax: +86 28 84617082,

† These authors contributed equally to this work.

Supplementary Table

Table 2 The ingredients of the normal chow diet (NCD).

**Macronutrient**	**gm%**	**kcal%**
Protein	20.4	20
Carbohydrate	65.2	70
Fat	5.3	10
Total	---	100
**Ingredient**	**gm**	**kcal**
Casein, 30 mesh	200	800
L-cystine	3	12
Corn starch	315	1260
Maltodextrin 10	35	140
Sucrose	350	1400
Microcrystalline cellulose	50	0
Soybean oil	25	225
Lard	20	180
V10001 mixed vitamin.	10	40
Total	1008	4057

Table 2 The ingredients of the high fat diet (HFD).

Macronutrient	gm%	kcal%
Protein	24	20
Carbohydrate	41	35
Fat	24	45
Total	---	100
**Ingredient**	**gm**	**kcal**
Casein, 30 mesh	200	800
L-cystine	3	12
Corn starch	72.8	291
Maltodextrin 10	100	400
Sucrose	172.8	691
Microcrystalline cellulose	50	0
Soybean oil	25	225
Lard	177.5	1598
V10001 mixed vitamin.	10	40
Total	811.1	4057

© 2018 by the authors. Submitted for possible open access publication under the terms and conditions of the Creative Commons Attribution (CC BY) license (http://creativecommons.org/licenses/by/4.0/).
